# Seasonal and spatial dynamics of the microbiome of the polychaete *Lanice conchilega* in the Wadden Sea

**DOI:** 10.1038/s41598-025-25737-3

**Published:** 2025-10-28

**Authors:** Manuel Lanza Guedán, Mike Smykala, Simon Käfer, Jasmin S. Mueller, Kertu Lohmus, Daniela Pieck, Bert Engelen, Gabriele Gerlach

**Affiliations:** 1 Institute of Biology and Environmental Sciences, Carl von Ossietzky Universität Oldenburg, Ammerländer Heerstraße 114-118, 26111 Oldenburg, Germany; 2 Helmholtz Institute for Functional Marine Biodiversity Oldenburg (HIFMB), Im Technologiepark 5, 26129 Oldenburg, Germany; 3 Institute for Chemistry and Biology of the Marine Environment (ICBM), Carl von Ossietzky Universität Oldenburg, Ammerländer Heerstraße 114-118, 26111 Oldenburg, Germany; 4 Senckenberg am Meer, Department for Marine Research, Südstrand 40, 26382 Wilhelmshaven, Germany

**Keywords:** Microbiome, 16S rRNA gene amplicon sequencing, *L. conchilega*, Abiotic conditions, Eulittoral, Sublittoral, *Endozoicomonas* spp., Host-microbiome interactions, Environmental adaptation, Microbiology, Ocean sciences

## Abstract

Rapidly changing abiotic conditions, particularly temperature variations, pose adaptation challenges to many animal communities across the globe. *Lanice conchilega*, a key polychaete in the ecosystem of the German Wadden Sea, faces an increasing threat hampered by its limited mobility. The microbiome can alleviate stress, alter local environmental conditions, and provide new metabolic capabilities, contributing to host adaptation. To understand the potential roles of the microbiome of *L. conchilega*, we analysed a broad sample set via 16 S rRNA gene amplicon sequencing. We compared the bacterial composition of the microbiome of *L. conchilega* with that of the surrounding sediment, and the effect of seasonality, zonation, tidal, and diurnal cycles. Our findings reveal a set of ASVs exclusive to the microbiome of *L. conchilega*. Community composition was mostly shaped by seasonality, slightly influenced by zonation, day/night cycles, and tides. Notably, *Endozoicomonas* ASVs were found only in the *L. conchilega* microbiome and were particularly abundant during summer. These bacteria, known for their symbiotic relationships, broad metabolic capabilities, and linked to heat resistance in corals, are predicted to contribute unique metabolic functions in *L. conchilega*. This work provides novel insights into the host-microbiome relationship of *L. conchileg*a and highlights the potential role of symbionts in the environmental adaptation of these and similar animals.

**Supplementary Information:**

The online version contains supplementary material available at 10.1038/s41598-025-25737-3.

## Introduction

Many animal species, challenged by the unprecedentedly high speed of environmental changes, are unlikely to survive exclusively through evolution via genetic recombination and natural selection. For a long time now, it is known that the microbiome is essential for the host´s life in many organisms, resulting in a co-dependent organism termed “holobiont”^1^. Prokaryotes are a crucial component of these microbiomes; as they possess mechanisms for fast evolution, such as rapid replication, high mutation rates, and horizontal transfer. Consequently, in recent years, the prokaryotic part of the microbiome has become a target of extensive research efforts to gather a deeper understanding of fast-paced adaptation and evolution mechanisms.

These research efforts have yielded critical information about the potential of the microbiome, which has been upheld to be an evolution accelerator^2^. Microbiomes can alter host development and physiology^3^, enhance disease resistance^4^, increase the host´s tolerance to abiotic stressors^5,6^, and modulate niche size^7^. Host-associated bacteria (henceforth microbiome) can extend the adaptive plasticity of a host, offering new avenues for adaptation beyond the host’s inherent metabolic capabilities^8^. Rosado et al.^9^ showed that certain bacterial consortia can be used as probiotics to confer temperature and pathogen resistance to corals. Studies by Doering et al.^10^. and Baldassarre et al.^11^ in corals and anemones, respectively, showed that a shift in microbiome composition can confer high-temperature adaptation to the host. Thus, host-microbiome interaction appears to be an essential mechanism for animal adaptation, especially in scenarios with sharp changes in abiotic conditions. Despite the increasing efforts, the nature of host-microbiome relationships is very complex, and the impact of the microbiome on the host´s health is, in most cases, poorly understood.

The Wadden Sea is a unique environment highly threatened by the expected changes in abiotic conditions driven by climate change. Although the Wadden Sea has a long history of human alteration through activities such as fishing, agriculture, and diking^12–15^, protection efforts have intensified in recent years^16,17^ to preserve this unique environment, which was declared a UNESCO World Heritage Site in 2009. The Wadden Sea features large eulittoral areas, where marine organisms directly experience the tidal cycles by frequent inundation and desiccation. These rapid fluctuations lead to changing salinities, nutrient availability, and temperatures. On the other hand, sublittoral populations of the same species do not experience this high variability, and several studies have reported broader tolerance limits in eulittoral organisms^18–20^. Nonetheless, due to increasing water temperatures, populations from both areas might already live at the limit of their thermal tolerance^19^.

*Lanice conchilega* is a keystone species in the ecosystem of the Wadden Sea. These tube-building polychaetes create three-dimensional structures that modify the characteristics of their environment and thus are referred to as bioengineers^21^. This form of bioengineering provides several benefits, both *L. conchilega* and for its surrounding environment by increasing habitat heterogeneity^22^ and biodiversity^23^. As a result of this trait, adult individuals of this species live semi-sessile, showing only vertical migration inside the self-built tube, up to 40 cm long, to avoid adverse conditions or predators. This trait, in other ways advantageous, narrows their capability to escape potentially lethal abiotic conditions such as extreme heat. In addition, new regulations were introduced in the 1980s, leading to water de-eutrophication and consequent decrease of algal-bloom events. While beneficial in many respects, this reduction in nutrient levels likely decreased the biomass available to *L. conchilega*, since they feed on both deposited and suspended particles^24^. This is likely to be an important driver of a significant decline in the *L. conchilega* population and other macrofauna in the German Wadden Sea during the last few decades^25^.

So far, there is no information about the microbiome of *L. conchilega* or its potential contribution to the host´s fitness. Nonetheless, symbiotic interactions could play a critical role in the health and adaptation of *L. conchilega*, as they do in several marine organisms. Thus, acquiring host-specific information on the bacterial communities associated with *L. conchilega* is essential to underpin potential symbiotic relationships. Moreover, revealing how different abiotic conditions affect microbiome composition could shed light on the nature of the host-bacteria interactions and their contribution to the host. This knowledge might be critical in future decision-making, increasing the chances of survival for *L. conchilega*, and mitigating further loss of macrofauna.

Therefore, this work aims to gain insights into the bacterial community associated with *L. conchilega* and the effects of abiotic stressors on the microbiome composition via 16 S rRNA gene amplicon sequencing, an efficient method to study microbiome-host dynamics^26^.

## Methods

### Sampling

**Fig. 1:**
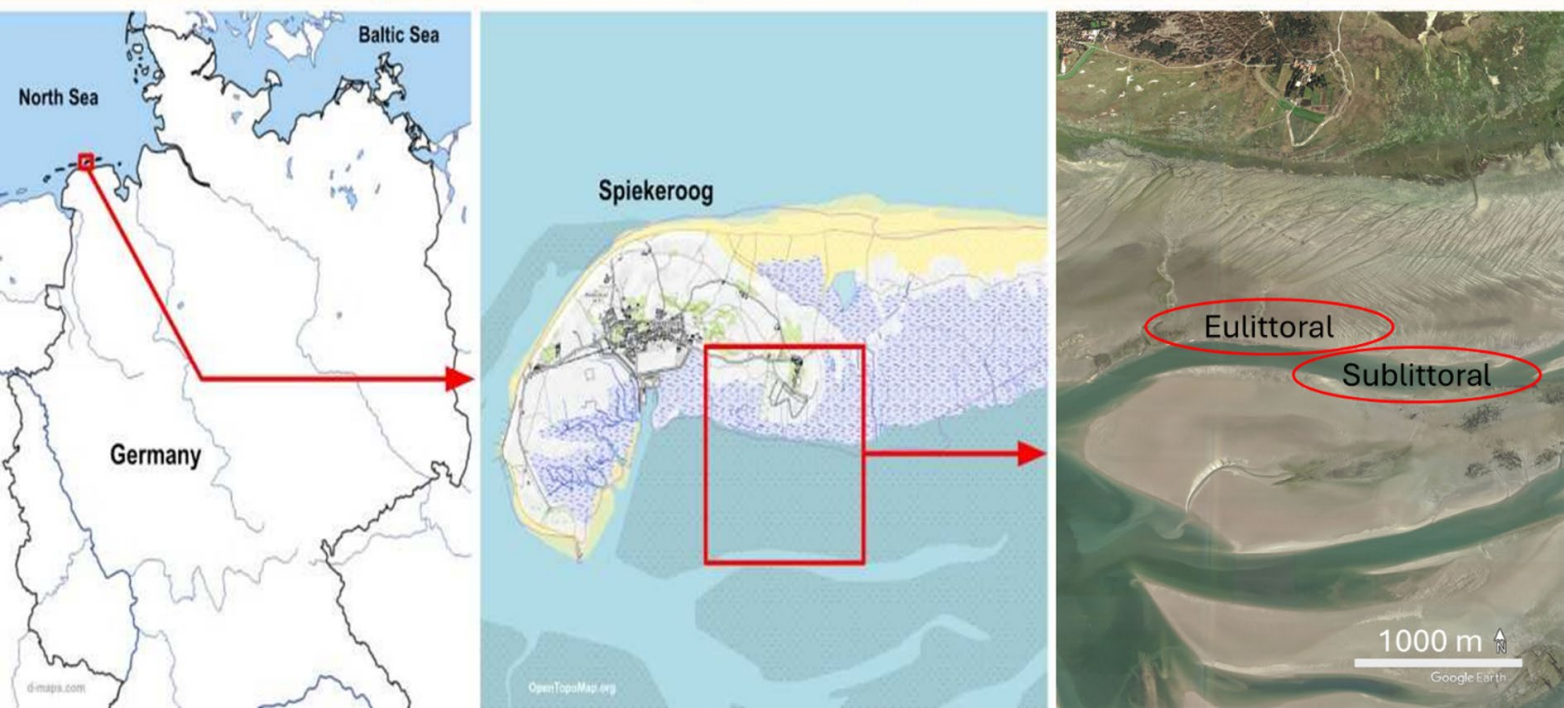
Geographic location of the study sites. The left panel highlights the position of Spiekeroog among the German Eastern Frisian Islands. The middle panel shows the eastern part of the island of Spiekeroog. The right panel shows the eulittoral and sublittoral sampling sites where *L. conchilega* and sediment samples were collected. Images (left to right) were created with www.d-maps.com, http://www.OpenTopoMap.org, and Google Earth.

In August of 2022 and May 2023, two sampling campaigns were conducted in the back barrier tidal flats of the East Frisian Island of Spiekeroog (Fig. 1). Samples were collected in the eulittoral and sublittoral zones. For each zone, 10 *L. conchilega* individuals were collected both day and night and at low and high tide, resulting in a total of 80 specimens. Low-tide samples were obtained by digging out sediment, approximately 30 cm deep, with a fork. During high tide and in the sublittoral zone, sediment was obtained with a box corer on board the research vessel R/V Otzum. Upon collection, all sampled individuals were killed using a lethal concentration of tricaine mesylate (MS-222) and directly embedded in RNA later. Samples were stored for 1 to 4 days at 4 °C and at -80 °C upon arrival at the university facilities.

Corresponding sediment samples were collected using 5 mL sterile syringes with the tip cut off to retrieve intact sediment cores. Upon collection, the syringes were sealed using Subda-Seal Silicon-Septa size 21 (Sigma/Merck, Darmstadt, Germany), cooled with ice packs in a cooler box, and stored at -20 °C until further use.

The full sample set of this study comprises 137 *L. conchilega* individuals from the North Sea off the tidal basin of Spiekeroog and 130 sediment samples from the surrounding sediment. Mudflat sediment (eulittoral) and water temperature (4 m depth, sublittoral) data were acquired in the Spiekeroog long-term measuring station^27^.

While aiming to acquire the same number of samples in the May 2023 sampling campaign, only 57 out of 80 samples were collected due to technical problems with the box corer when sampling the sublittoral area. Twenty-three samples of the sublittoral group were lacking in the data set from May. These could not be compensated for without sacrificing consistency with the remaining sampling campaign.

### Library preparation and sequencing

Every individual was cut into approximately 5 mm sections and embedded in a protective solution to preserve the genetic material (Protective reagent from Monarch^®^ Total RNA Miniprep kit). Cut tissue samples were beaten with 5 mm steel beads using the Tissuelyser II, QIAGEN. (2007) for 10 min. Sediment cores were divided into subsections of 0–1 cm, 1–2 cm, and 2–3 cm below the surface. 0.25 g of sediment was used for extraction. DNA was extracted using the DNeasy PowerSoil Pro kit, QIAGEN. (2019). The primers chosen to create the 16 S rRNA gene amplicon were the universal 515 F/926R (GTGYCAGCMGCCGCGGTAA / CCGYCAATTYMTTTRAGTTT) for the V4-V5 regions^28^. PCR settings and reagents are specified in the supplementary material (Tables 1 and 2). PCR products were checked via agarose gel electrophoresis (1.5%), quantified using a Qubit dsDNA HS kit from ThermoFischer Scientific, and purified using the SequalPrep^tm^ Normalization Plate kit from Invitrogen (Catalog no: A10510-01). After purification, DNA concentrations were again measured, normalized, and diluted to equal concentrations (10 nm). Pooled PCR products were sent for sequencing to the facilities at the Cologne Center for Genomics (CCG, Illumina MiSeq 2 × 300 bp). Sediment samples followed the same library preparation procedure and were sequenced in the ICBM, University of Oldenburg (NexSeq 2 × 300 bp).

### Read processing and data analysis

Raw reads were demultiplexed (bcl2fastq; https://emea.support.illumina.com/sequencing/sequencing_software/bcl2fastq-conversion-software.html) and processed as described in the default Fuhrmann pipeline for 515 F and 926R 16 S universal primers^29^. (https://github.com/jcmcnch/eASV-pipeline-for-515Y-926R). All steps were performed in a standardized Conda environment to increase reproducibility (qiime2-2022.2-DADA2-SILVA138.1-PR2_4.14.0). Qiime2^30^ was used for quality filtering of sequences. Primer sequences were removed using Cutadapt^31^ (20% of primer sequence mismatch allowed). 16 S rRNA sequences were isolated with the “bbtools” package^32^ and SILVA138^33^. Low-quality ends (quality score < 30) were trimmed (Forward sequences at 220 bp and reverse sequences at 200 bp), and DADA2^34^ was used to denoise, merge, and remove chimeras. Taxonomic data was assigned to each sequence with the Qiime2 classify-sklearn plugin, with SILVA138 and PR2 as the reference databases^33,35^. For the following analyses, chloroplast and mitochondrial sequences were excluded.

R 4.3.3^36^ was used to process the ASV table, obtain a taxonomic profile, and perform all analyses. Rarefaction curves (supplementary material, Figs. 1 and 2) were performed using the “Phyloseq” package version 1.44.0^37^. Total sum scaling to the median (38.912) was done to account for differences in sequencing depth. From the resulting 39.172 unique and classified ASVs, 868 (2.2%) were not classified further than the Phylum level. The “vegan” package^38^ was used for biodiversity and statistical purposes, and “ggplot2” version 3.5.1^39^ for plotting results.

To assess α-diversity, we used Shannon, Inverse Simpson (Dominance index), and Chao1. We performed an ANOSIM and a pairwise comparison with Tukey Honest Significance (HSD). Beta diversity was evaluated to understand differences in community composition. For this purpose, NMDS and PERMANOVA analyses were performed on Bray-Curtis dissimilarity community matrices. To gather further information about which taxa are relevant to group transitions, we used SIMPER analyses and then examined the changes in the relative abundance of the highlighted taxa.

Functional pathway abundances were predicted using PICRUSt2 (Phylogenetic Investigation of Communities by Reconstruction of Unobserved States)^40^. The ASV table was normalized before prediction, and the pipeline was executed with the “stratified” option to analyze the individual contribution of each ASV to the overall pathway abundance. The normality of the distribution of the abundance was checked with the Shapiro test and the package “ALDEx2”^41^, and “ggpicrust2”^42^ were used to test the differences between the abundances of every pathway. For pathways with normal distribution, the Welch t-test was applied. For pathways with non-normal distribution, the Wilcoxon rank test was applied. The predicted pathways were manually annotated using Metacyc (MetaCyc: Metabolic Pathways From all Domains of Life).

## Results

To investigate the microbial relationships of *L. conchilega*, we analyzed their microbiome across different natural environmental conditions. The collected samples (Table 1) represented five different variables, including microbiomes of *L. conchilega* and surrounding sediment in four abiotic conditions: season (summer vs. spring), zone (eulittoral vs. sublittoral), tide (high tide vs. low tide), and time (day vs. night). 57,811 unique ASVs from 225 classes, 574 orders, 998 families, and 1717 different genera were identified.

**Fig. 2:**
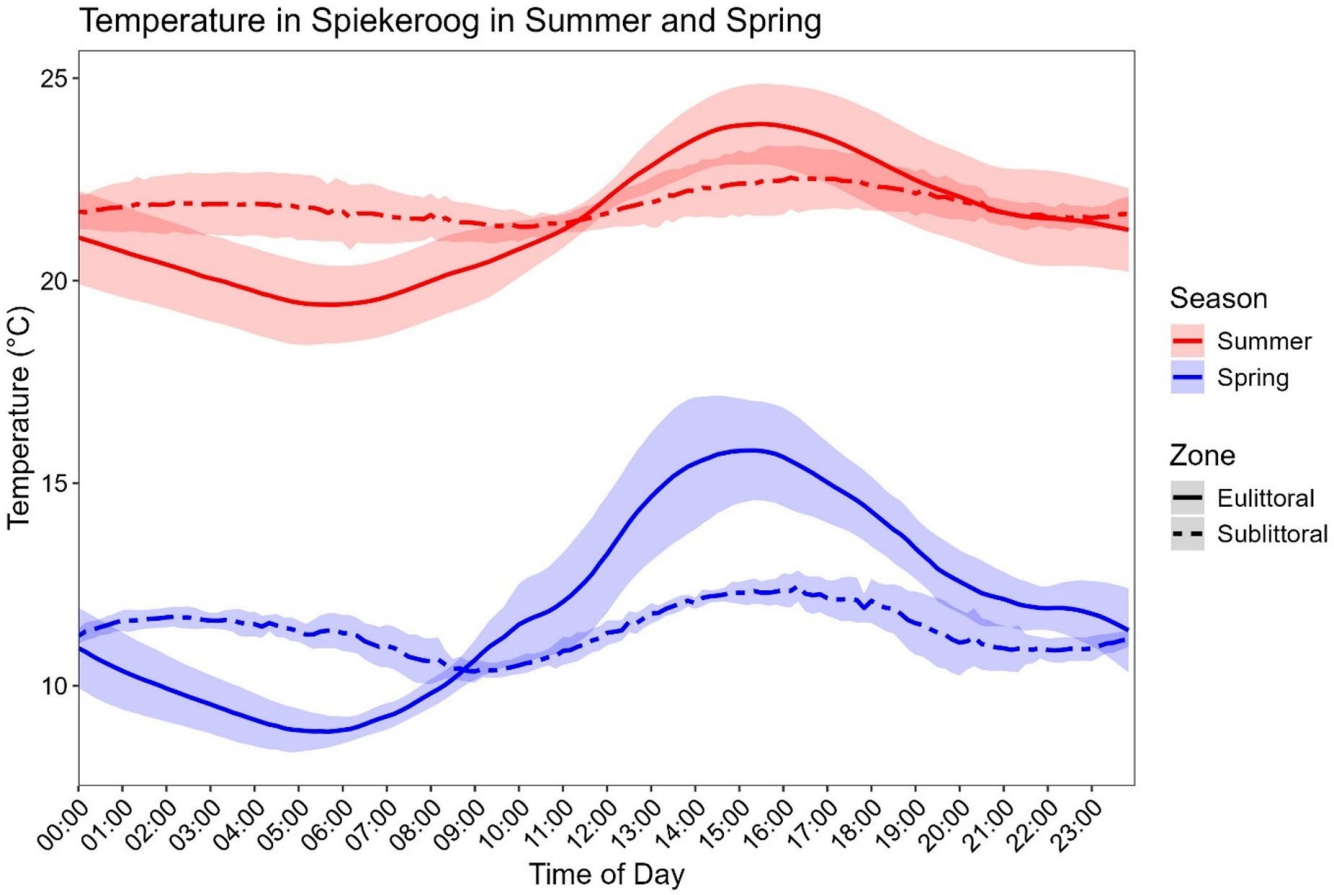
Temperature in Spiekeroog during the sampling campaigns. Mean temperature (± SD) recorded in the eulittoral (surface sediment temperature) and sublittoral (water column temperature, 4 m deep) zones during sampling in spring (blue) and summer (red). Solid lines represent the eulittoral zone, while dashed lines represent the sublittoral zone. Shaded regions indicate standard deviation.

Temperatures in August 2022 ranged from 20 °C at night (20.3 ± 1.2) to 25 °C during the day (22.3 ± 1.4) in the eulittoral, while in the sublittoral, temperatures remained constant between 21 °C (Night; 21.7 ± 0.6) and 22 °C (Day; 21.9 ± 0.6). In May, temperatures in the eulittoral had the highest variance, oscillating around 10 °C at night (10.0 ± 1.2), going up to 17 °C (13.4 ± 2.0). Sublittoral temperatures in May were stable at 11 °C (Night: 11.3 ± 0.4; Day: 11.4 ± 0.8) (Fig. 2).

**Table 1:** Overview of microbiome samples collected across season, zone, time, and tide.

Microbiome origin	Season	Zone	Time	Tide	*N*
*L. conchilega*	Summer	Eulittoral	Day	High	10
*L. conchilega*	Summer	Eulittoral	Day	Low	10
*L. conchilega*	Summer	Eulittoral	Night	High	10
*L. conchilega*	Summer	Eulittoral	Night	Low	10
*L. conchilega*	Summer	Sublittoral	Day	High	10
*L. conchilega*	Summer	Sublittoral	Day	Low	10
*L. conchilega*	Summer	Sublittoral	Night	High	10
*L. conchilega*	Summer	Sublittoral	Night	Low	10
*L. conchilega*	Spring	Eulittoral	Day	High	10
*L. conchilega*	Spring	Eulittoral	Day	Low	10
*L. conchilega*	Spring	Eulittoral	Night	High	10
*L. conchilega*	Spring	Eulittoral	Night	Low	10
*L. conchilega*	Spring	Sublittoral	Day	High	7
*L. conchilega*	Spring	Sublittoral	Day	Low	10
Sediment	Summer	Eulittoral	Day	High	9
Sediment	Summer	Eulittoral	Day	Low	9
Sediment	Summer	Eulittoral	Night	High	9
Sediment	Summer	Eulittoral	Night	Low	9
Sediment	Summer	Sublittoral	Day	High	9
Sediment	Summer	Sublittoral	Day	Low	9
Sediment	Summer	Sublittoral	Night	High	8
Sediment	Summer	Sublittoral	Night	Low	9
Sediment	Spring	Eulittoral	Day	High	9
Sediment	Spring	Eulittoral	Day	Low	7
Sediment	Spring	Eulittoral	Night	High	8
Sediment	Spring	Eulittoral	Night	Low	9
Sediment	Spring	Sublittoral	Day	High	8
Sediment	Spring	Sublittoral	Day	Low	9
Sediment	Spring	Sublittoral	Night	Low	9

### Is the *L. conchilega* microbiome different from the sediment´s microbiome?

**Fig. 3:**
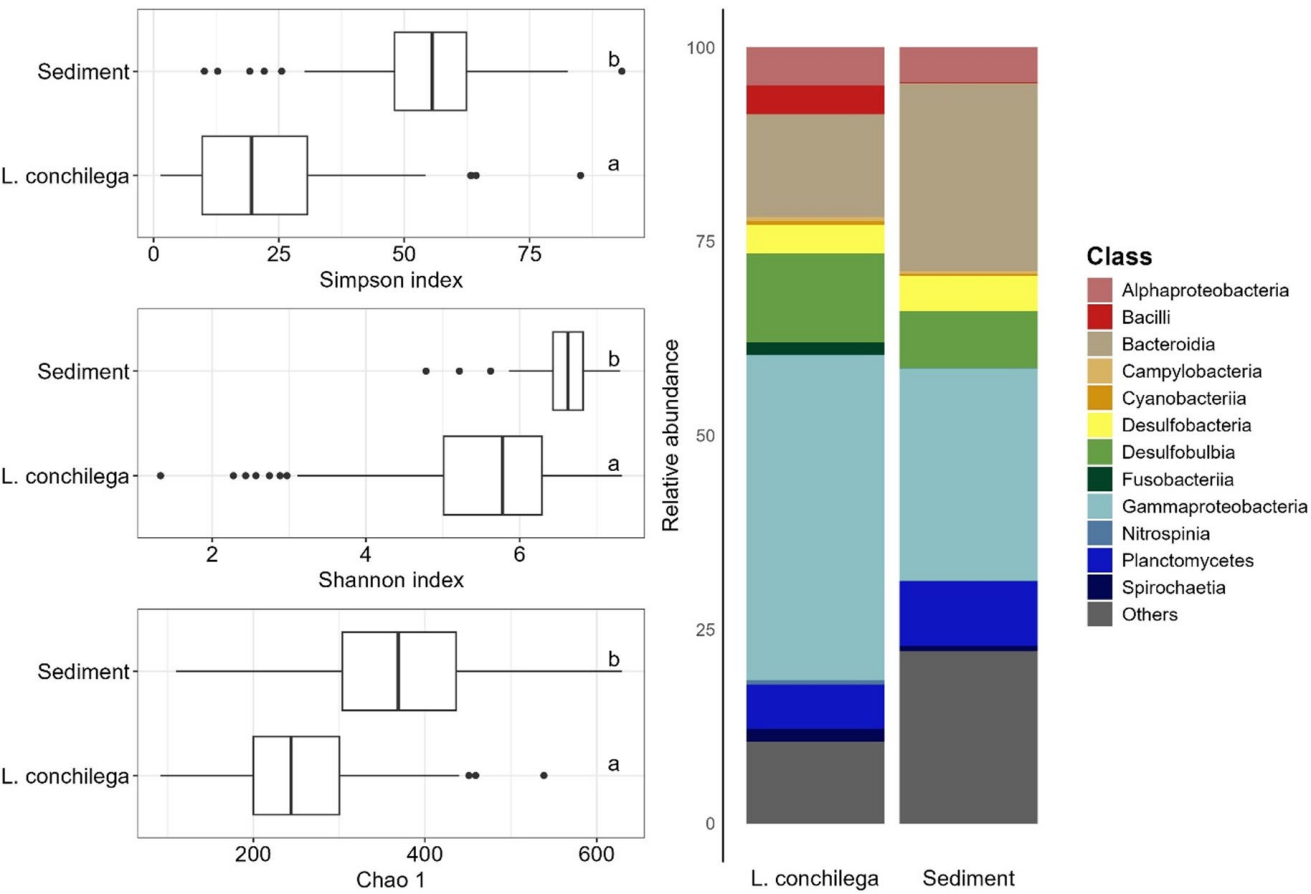
Origin Comparison of Alpha Diversity and Community Composition. Left: Boxplots showing Simpson, Shannon, and Chao1 indices comparing all sediment and *L. conchilega* microbiomes. The bold horizontal line indicates the median, the top and bottom of the box correspond to the 75th and 25th percentiles, and the whiskers extend to the largest and smallest values within the 1.5 interquartile range; points outside this range represent outliers. Letters (a, b) to the right of the boxes indicate statistical significance. Right: Bar plots showing the average relative abundance of every class with ≥ 10% relative abundance in at least one sample.

The alpha diversity of the *L. conchilega* microbiome (calculated at the genus level, supplementary material, Table 3) differed substantially from that of the sediment microbiome. Species richness, measured by the Chao1 index, was significantly lower in the *L. conchilega* microbiome (median = 244) compared to the sediment (median = 369; ANOVA: F = 95, *p* < 0.001, η² = 0.28). Similarly, both Shannon (ANOVA: F = 107.71, *p* < 0.001, η² = 0.31) and Inverse Simpson indices (ANOVA: F = 306.11, *p* < 0.001, η² = 0.56) were considerably lower in the *L. conchilega* microbiome than in the sediment. These results confirm that the observed *L. conchilega* microbiome is characterized by a smaller community with higher dominance of a few species, in contrast to the more even and diverse microbiome inhabiting the surrounding sediment (Fig. 3, left).

The *L. conchilega* microbiome was characterized (Fig. 3, right) by a high content of Gammaproteobacteria (42.1 ± 16%), followed by Bacteroidia (13.3 ± 10.8%) and Desulfobulbia (11.1 ± 5.6%). The most abundant classes in the sediment bacterial community were Gammaproteobacteria (27.54 ± 5.1%), Bacteroidia (24.3 ± 9%), and Planctomycetes (8.3 ± 2.1%). Several taxa were found uniquely in the bacterial community of *L. conchilega*, including unique Gammaproteobacteria, despite the large portion of Gammaproteobacteria in the sediment bacterial community. In *L. conchilega*, the most abundant Gammaproteobacteria were *Endozoicomonas* (37.7%), *Halioglobus* (14.6%), and *Vibrio* (7.9%).

**Fig. 4:**
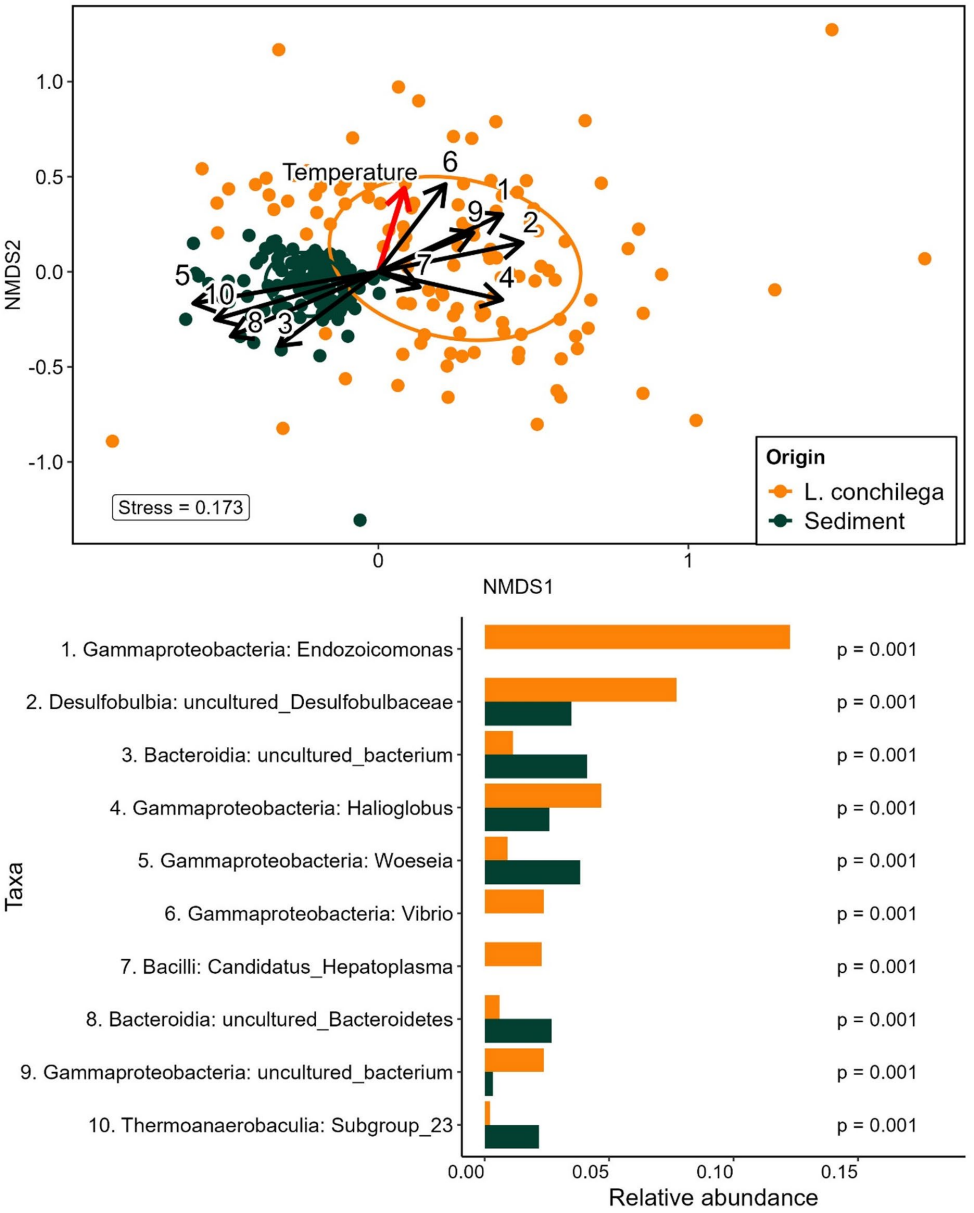
NMDS Ordination and Key Taxa Driving Origin Dissimilarity by SIMPER. Top: Non-metric multidimensional scaling (NMDS) plot based on a Bray-Curtis dissimilarity matrix for all *L. conchilega* and Sediment samples. In orange, *L. conchilega* microbiome, in dark green, sediment microbiome. Black arrows indicate the fitted environmental vectors of the top 10 taxa contributing most to dissimilarity (full taxon names shown below). The red arrow represents the fitted vector for temperature. Bottom: Relative abundance of the top 10 SIMPER-identified taxa contributing to dissimilarity between *L. conchilega* and sediment microbiomes. P-values from permutational tests are shown on the right.

PERMANOVA analysis on the Bray-Curtis dissimilarity matrix showed that the origin of the samples (*L. conchilega* vs. sediment, supplementary material, Table 5) explained 24.5% of the differences between the groups (F_1, 239_ = 77.7, R^2^ = 0.245, *p* < 0.001, 10000 permutations). The Bray-Curtis dissimilarity-based NMDS plot (Fig. 4, top) helps highlight these differences as the different microbiomes cluster together. Intragroup variation was significantly higher in *L. conchilega* than in the sediment microbiome, as assessed by Beta Dispersion Analysis. The distance to the median point in *L. conchilega* and sediment samples was 0.417 and 0.245, respectively (*p* < 0.001).

The results from the SIMPER analysis (Fig. 4, bottom) show the importance of several taxa as the main drivers of intergroup dissimilarities. The top contributors to the dissimilarity at genus level between *L. conchilega* microbiome and sediment microbiome were ASVs belonging to *Endozoicomonas*, Desulfobulbia: uncultured *Desulfobulbaceae*, uncultured Bacteroidia, *Halioglobus*, and *Woesia*, among many others, which showed a significant difference between the two groups (permutational test, *p* < 0.001, Fig. 1, D). We highlight *Fusobacteraceae*, which was not among the top 10 contributors to the observed dissimilarities but was significantly more abundant in *L. conchilega* and very scarce in the sediment microbiome (1.6% in *L. conchilega* vs. < 0.001% in sediment, *p* < 0.001).

After removing every genus present in the sediment microbiome from the *L. conchilega* microbiome, 419 genera remained, which were found exclusively in the organism´s microbiome. The most abundant taxon in the remainder microbiome was *Endozoicomonas spp.* (*Endozoicomonadaceae*), representing around 60% of the remaining ASV read counts, followed by two unclassified strains of Firmicutes (9.4% and 2.8%). Only 7 out of 224 remaining taxa had a mean abundance > 0.01%.

### How do the abiotic conditions affect microbiome composition?

The size effect and statistical significance of every variable in our sample set on the *L. conchilega* microbiome composition were calculated via PERMANOVA analysis. We observed a statistically significant effect of every variable in this work, even though the magnitude of these impacts differed (Supplementary material, Figs. 3, 4 and 5). The effect of these variables proved to be larger on the sediment community than on the *L. conchilega* community. The largest impact of the explored conditions on the *L. conchilega* microbiome was found in seasonality (supplementary material, Table 5) (F_1, 112_ = 10.71, R^2^ = 0.080, *p* < 0.001), followed by zonation (F_1, 112_ = 4.82, R^2^ = 0.039, *p* < 0.001), tide (F _1, 112_= 3.30, R^2^ = 0.024, *p* < 0.001), and lastly diurnal cycles (F_1, 112_ = 2.44, R^2^ = 0.018, *p* < 0.01). On the other hand, the largest impact of the explored conditions on sediment microbiome was found in the zonation (supplementary material, Table 6)(F_1, 119_ = 20.13, R^2^ = 0.118, *p* < 0.001), followed by seasonality (F_1, 119_ = 16.55, R^2^ = 0.097, *p* < 0.001), time (F_1, 119_ = 5.83, R^2^ = 0.034, *p* < 0.001), and lastly, tide (F_1, 119_, = 5.08, R2 = 0.029, *p* < 0.001).

### Effect of seasonality on the *L. conchilega* microbiome

To allow a fair comparison between the microbiomes of *L. conchilega* in different seasons, only eulittoral samples were used, since many sublittoral samples were absent in the spring dataset.

**Fig. 5:**
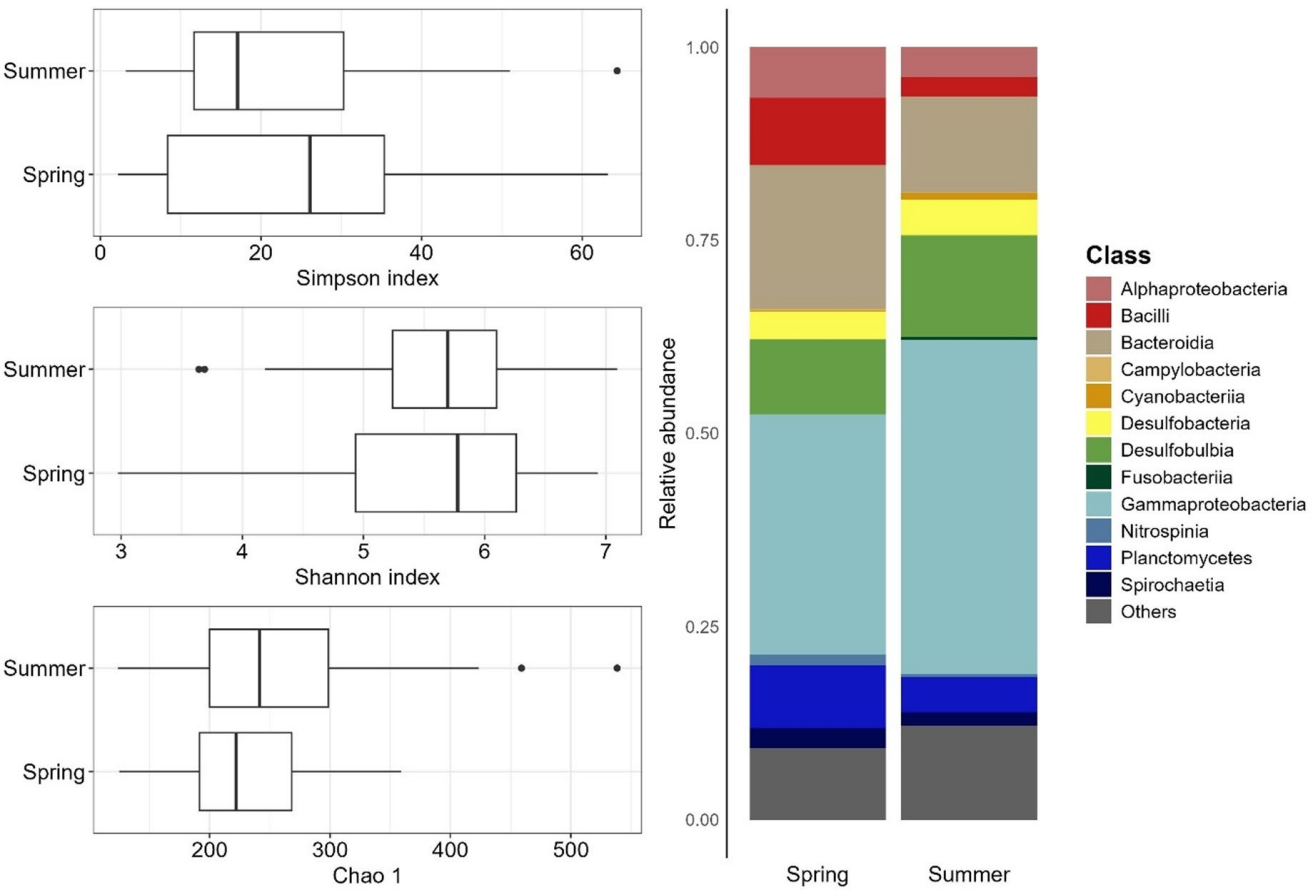
Seasonal comparison of alpha diversity and community composition in *L. conchilega* microbiomes. Left: Boxplots showing Simpson, Shannon, and Chao1 indices comparing eulittoral *L. conchilega* microbiomes in spring and summer. The bold horizontal line indicates the median, the top and bottom of the box correspond to the 75th and 25th percentiles, and the whiskers extend to the largest and smallest values within the 1.5 interquartile range; points outside this range represent outliers. Right: Bar plots showing the average relative abundance of every family with ≥ 10% relative abundance in at least one sample.

Seasonality significantly influenced the composition of the *L. conchilega* microbiome, even if alpha diversity did not differ significantly between summer and spring (Fig. 5). The total number of species, assessed by Chao1, was slightly higher in summer samples compared to spring samples (supplementary material, Table 7; median: 253 vs. 222; ANOVA: F = 1.99, *p* = 0.16, η² = 0.031). Simpson index in summer samples displayed lower values than spring samples (median: 17.22 vs. 26.57; ANOVA: F = 0.82, *p* = 0.37, η² = 0.013). These results indicate a similar diversity, but hint at a change in microbiome composition in summer towards a more uneven community.

**Fig. 6:**
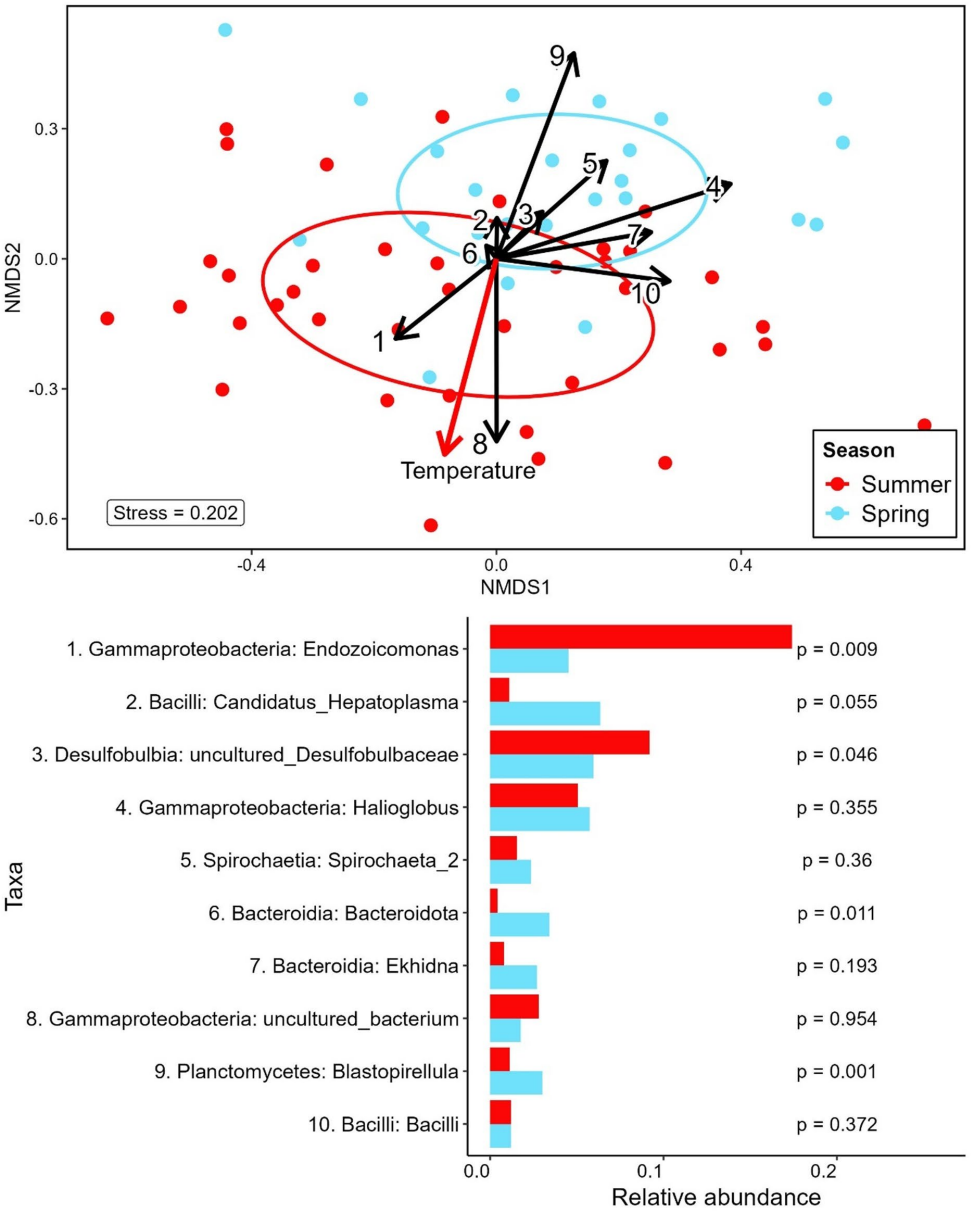
NMDS Ordination and Key Taya Driving Seasonal Dissimilarity by SIMPER. Top: Non-metric multidimensional scaling (NMDS) plot based on a Bray-Curtis dissimilarity matrix for eulittoral *L. conchilega* samples. In red, summer samples; in light blue, spring samples. Black arrows indicate the fitted environmental vectors of the top 10 contributing most to dissimilarity (full taxon names shown below). The red arrow represents the fitted vector for temperature. Bottom: Relative abundance of the top 10 SIMPER-identified taxa contributing to dissimilarity between summer and spring eulittoral *L. conchilega* microbiomes. P-values from permutational tests are shown on the right.

The differences in composition are observable in the NMDS plot (Fig. 6, top). The SIMPER analysis (Fig. 6, bottom) highlights ASVs belonging to taxa such as *Endozoicomonas*, Candidatus *Hepatoplasma*, *Desulfobulbaceae*, and *Halioglobus* as the major drivers of this change. *Endozoicomonas* represents the largest microbiome dissimilarity contribution, accounting for 16% in summer samples and 5% in spring samples. This significantly higher relative abundance of *Endozoicomonas* (permutational test; *p* < 0.01) in summer samples is the major factor contributing to seasonal dissimilarities observed in the eulittoral *L. conchilega* microbiomes. *Hepatoplasma* is highlighted as the second group with the most contribution to seasonal dissimilarity and were more abundant in spring (6.4 ± 1.8% in spring vs. 1.1 ± 0.2% in summer).

**Fig. 7:**
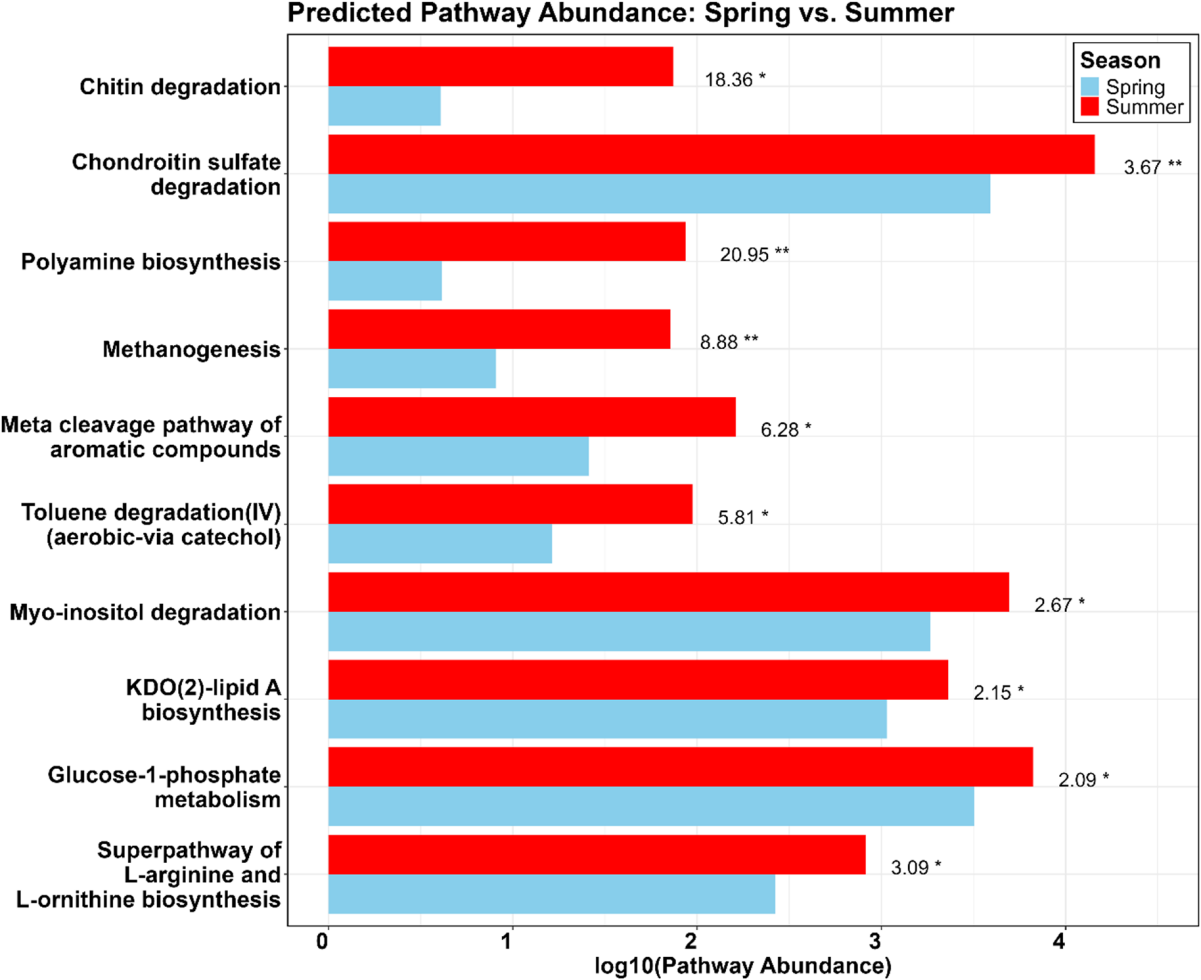
Predicted Pathway Abundance in *L. conchilega* Microbiomes during Spring and Summer. Bar plot showing the log₁₀-transformed predicted abundances of the top 10 metabolic pathways with a fold change (FC) ≥ 2. Red bars represent summer samples, and blue bars represent spring samples. Values to the right represent FC and asterisks show statistical significance (*p* < 0.05: *, *p* < 0.01: **).

The functional prediction of the genetic potential of the bacterial communities using PICRUSt2 revealed that several predicted pathways are significantly more abundant in summer samples. We calculated the fold change (FC) of pathway abundance between summer and spring to explore which predicted functions might reflect ecologically relevant changes. The most abundant predicted pathways with FC > 2 were chondroitin sulfate degradation, glucose-1-phosphate metabolism, and Myo-inositol degradation. The predicted pathways with the highest FC were polyamine biosynthesis, chitin degradation, and methanogenesis (Fig. 7). Notably, ASVs of *Endozoicomonas* represented a major contribution to some of these pathways, especially for chondroitin sulfate degradation, and chitin degradation, to which *Endozoicomonas* contributed 96.6% of the total counts in summer vs. 57.4% in spring, and 31.5% in summer vs. 12.2% in spring, respectively (supplementary material, Fig. 6).

## Discussion

### *Lanice conchilega* harbors a distinct microbiome

The microbiome of *L. conchilega* showed a relatively high taxonomic abundance, with ASVs belonging to a few hundred bacterial families. Nonetheless, there was a clear dominance of a few taxa. Many of the most abundant genera in *L. conchilega* were part of the sediment community, belonging to the families *Arenicellaceae*, *Cyclobacteriaceae*, *Desulfocapsaceae*, *Desulfosarcinaceae*, *Flavobacteriaceae*, *Haliaceae*, *Pirellulaceae*, and *Rhodobacteraceae.* These taxa are part of the microbiome of many previously described marine sediments, including the Spiekeroog mudflats^43^. These taxa are likely either enriched, thriving in commensalism, or detrimentally affected by the characteristics of the gut of *L. conchilega*. Other taxa in the *L. conchilega* microbiome are usually found in seawater bacterial communities, such as *Halioglobus*, *Rhodobacteraceae*, and *Pseudoalteromonas*.

SIMPER results suggested the importance of several taxa, such as *Endozoicomonas*, Entomoplasmatales: Candidatus *Hepatoplasma*, and *Fusobacteraceae*, which contributed highly to intergroup dissimilarity. *Fusobacteraceae* ASVs were not among the top 10 contributors to the dissimilarities via SIMPER analysis. However, *Fusobacteraceae* are significantly more abundant in *L. conchilega* than in the sediment microbiome (1.59% in *L. conchilega* microbiome vs. 0.0021% in sediment microbiome). *Fusobacteraceae* have been previously found in the microbiome of the polychaete *Hydroides elegans*^44^, but the nature of the relationship is unclear. Furthermore, this taxon is not commonly found in seawater; therefore, it is likely that it either engages in some symbiotic relationship with *L. conchilega* or is enriched by the conditions created by the host.

As their name suggests, Entomoplasmatales have been found in, and are known for their association with insects, where they have been described as both intra and extracellular symbionts^45,46^. These bacteria have been previously reported in the gut of shrimps and are absent in the surrounding environment^47^. In our study, *Hepatoplasma* was found in most of the analysed *L. conchilega* microbiomes and was more abundant in spring than in summer. Even though the pairwise comparison of SIMPER shows a marginally non-significant contribution for this bacterial taxon (*p* = 0.055), its contribution could be biologically relevant. This genus has been found in other macrozoobenthic organisms, such as isopods, shrimp, and crabs^48–50^, and possibly has some kind of role in symbiosis with their host. The size of their genome is relatively small (~ 650 kb), with reduced metabolic pathways, which could imply metabolic dependence from the host, engaging in commensalism, or perhaps providing some defensive mechanism against pathogens^51–53^.

A sizable part of the surrounding sediment community is comprised of sulfur-reducing bacteria, such as *Desulfocapsaceae* (Enriched in our *L. conchilega* microbiome) and *Desulfosarcinaceae*. These bacteria produce hydrogen sulfide under anoxic conditions. Therefore, it is likely that a bacterial community capable of recycling all these sulfur compounds is essential or very beneficial for *L. conchilega.*

The high abundance of *Vibrio* spp. observed in the *L. conchilega* contributed significantly to the dissimilarities with the sediment (*p* < 0.001). However, the presence of *Vibrio* in the *L. conchilega* microbiome must be considered cautiously. The occurrence and abundance of these bacteria were highly skewed. The importance of *Vibrio* was highly affected by a very high abundance in a few samples belonging to the sublittoral, high tide, night group. Due to the ecology of these marine bacteria and the found distribution in our samples, it is difficult to tell if the observed high abundance in a few samples is the result of either infectious processes or contamination.

The 16 S rRNA gene amplicons analyzed here reflect the bacterial composition of every tissue of *L. conchilega*. Therefore, the microbiome comprises bacteria inside the intestines, adhering to the epidermis, forming consortia inside a particular tissue, or even intracellularly. Due to their bottom-feeding behaviour, sediment-associated bacteria were expected to be found in the organism´s gut and outer surface. The comparison between the *L. conchilega* and the sediment microbiomes provides a strong basis to identify potential symbiotic or selectively enriched taxa within the organism. Once the taxa from the sediment were subtracted from the microbiome of *L. conchilega*, apart from *Endozoicomonas* (~ 60%), only a few taxa remained with an abundance over 1%, but over 400 genera remained with very low relative abundance. Some of these low-abundance ASVs could represent taxa from the seawater community. However, despite ingesting the surrounding sand and mud particles for nutrition, the *L. conchilega* microbiome does not mirror the sediment microbiome.

### Microbiome variability and abiotic influence

The differences found between sediment and *L. conchilega* microbiomes are partially explained by the variables explored in our study. The origin of around 83% of the observed *L. conchilega* bacterial community variation remains unexplained. Higher variability between *L. conchilega* microbiomes and a stronger effect of the studied variables on sediment microbiome composition suggest that the effects shaping the organism´s microbiome are far more complex. Following the intricacies of the microorganism-host relationship and host characteristics, *L. conchilega* imposes selective pressures on its microbiome. These pressures create a different environment that leads to a more specialized microbial community where symbiotic bacteria, either mutualistic or commensal, are enriched. In *L. conchilega*, such selective pressures can be exerted as physical barriers, such as their self-built tubes, skin, and mucus. Similarly, the decorator worm *Diopatra cuprea*, another eulittoral, tube-building polychaete, harbored a bacterial community within its tubes that significantly differed from the surrounding sediment (F_24,1_ = 4.0, R2 = 0.14, *p* = 0.001)^54^.

Other selective pressures may include immune interactions^55^, the life stage of the individuals^44,56^, and shifts in chemical composition, as oxygen availability and organic carbon concentration are the major drivers of marine sediment community composition^57^. Ma et al. (2023) analyzed the microbiome in the flatworm *Macrostomum lignano*, which belongs to the sand meiofauna of the Adriatic Sea and occurs within the eulittoral zone. They found that different developmental stages each harbor a specific microbiota, and that this microbiome exhibited circadian rhythmicity.

Overall, there is a common microbiome composition of *L. conchilega*, which fluctuates differently across all the studied variables, especially with seasonality. The higher dominance of *Endozoicomonas* in summer samples represents the biggest change across the whole data set of *L. conchilega* microbiome.

### Endozoicomonas as a potential mutualistic symbiont

*Endozoicomonas* spp. ASVs represented the most abundant genus, accounting for 12.65% of the total reads at the genus level in the microbiome associated with *L. conchilega* (average across all samples). This genus is responsible for the highest contribution to the dissimilarity, both between the *L. conchilega* microbiome and the sediment microbiome (absent in the latter), and between the summer and spring microbiomes of *L. conchilega*.

*Endozoicomonas* were originally found in sea slugs and described by Kurahashi and Yokota (2007)^58^ and found to engage in pathogenic processes^59^. Their ubiquity inside marine invertebrates, including a few polychaetes^60,61^ and a large genetic arsenal, suggested the possibility of diverse lifestyles^62^. The potential of *Endozoicomonas* as an essential part of the holobiont in corals has given this genus increasing attention in recent years, with other studies pointing to symbiotic roles in other marine invertebrates such as sponges and ascidians^3,63,64^. Recent research provides robust evidence of a mutualistic endosymbiotic lifestyle in corals, with highly specific strain-species relationships^65^. *Endozoicomonas* has been linked to coral thermotolerance, decreasing in abundance inside when bleaching occurs and increasing during recovery episodes^66–71^. These bacteria form intimate associations within the coral´s tissue, forming aggregates adjacent to *Symbiodinium* cells inside the organism´s endoderm^72,73^.

*Endozoicomonas* possess large genomes (5-7mb), with the potential to metabolize chitin and potentially provide their host with essential amino acids, vitamins, nitrogen compounds, and antimicrobial compounds^62,71,73–76^. In addition, these bacteria may play a role in the cycling of organosulfur compounds. Coral-associated *Endozoicomonas* contain the dddP gene for the DddP lyase^65^, which has been speculated to enable the successful bacterial colonization of different coral species^77,78^. The DddP lyase cleaves DMSP (Dimethylsulfoniopropionate), converting this molecule into DMS (Dimethylsulfide) and acrylate^79^. DMSP has been described as an osmolyte, providing tolerance to salinity changes^80^. However, DMSP production is likely to be upregulated under different kinds of stress and helps by scavenging reactive oxygen species (ROS)^81,82^. Therefore, efficient regulation of DMSP concentrations could play an essential role in the microbiome of such marine invertebrates^83^. This role would be increasingly important in eulittoral areas, where salinity is subject to abrupt fluctuations. The products of DMSP cleavage, DMS and acrylate are essential for local interactions. DMS is a volatile organosulfur molecule involved in many processes, such as cueing for predators and attracting organisms that could represent nourishment for the host^84^. Acrylate, on the other hand, serves as a carbon source.

Furthermore, in *L. conchilega*,* Endozoicomonas* ASVs accounted for 16.5% of the total community in summer samples and 5% in spring samples. This difference in abundance in *Endozoicomonas* represents the main driver of the differences observed between seasons in the *L. conchilega* microbiomes. This switch in composition may be the product of a dynamic relationship of the host with these specific bacteria, which could contribute to temperature adaptation or represent an advantage, providing specific metabolic role capabilities, such as chitin degradation and chondroitin sulfate degradation. These functions have been predicted here to be much more abundant in the summer *L. conchilega* microbiome, and this change in the predicted functions has been attributed mainly to the contribution of genes belonging to *Endozoicomonas*. Thus, a higher abundance of these bacteria may represent an important metabolic advantage, especially when facing nutrient scarcity or an increased metabolic rate driven by abiotic stressors such as temperature. Because of the well-documented symbiosis between *Endozoicomonas* and corals, its presence in other closely related organisms, and the results found here, we suggest that *Endozoicomonas* spp. lives in symbiosis with *L. conchilega*, potentially engaging in a mutualistic relationship and helping in their adaptation.

Current microbiome stewardship efforts have largely focused on iconic reef-building organisms like corals. Different approaches to their conservation have been extensively discussed to avoid massive bleaching and improve their resilience. In recent years, assisted evolution has been proposed as a potentially feasible, effective tool to help corals cope with extreme temperature events^85,86^.

This concept could be transferable to other marine ecosystem engineers. *Lanice conchilega*, similarly, plays a foundational role in the benthic ecosystem of the Wadden Sea and other temperate regions by altering sediment structure and enhancing biodiversity. The presence of *Endozoicomonas*, a genus frequently associated with mutualistic relationships in marine invertebrates in the microbiome of *L. conchilega*, suggests functionally relevant host-microbe associations. This opens a promising avenue to explore microbiome-based strategies for stress resilience and ecological restoration. Even though human intervention at this scale must be thoroughly studied and applied with severe caution, knowing the intricacies of the organism´s interactions with its microbiome would lay a foundation to work towards this goal^87^.

## Supplementary Information

Below is the link to the electronic supplementary material.

Supplementary Material 1

## Data Availability

The raw data supporting the conclusions of this article will be made publicly available by the authors under the.
