# Supplementary material for: Seasonal and spatial dynamics of the microbiome of the polychaete Lanice conchilega in the Wadden Sea
Source: Sci Rep. 2025 Oct 28;15:37731. doi: 10.1038/s41598-025-25737-3 (PMC12569044; doi:10.1038/s41598-025-25737-3)
Supplement: Supplementary file 1 — Supplementary Material 1 [file 41598_2025_25737_MOESM1_ESM.pdf]

Supplementary material

Figures

Figure 1. Rarefaction curves for all samples clustered by Origin (L. conchilega vs Sediment)

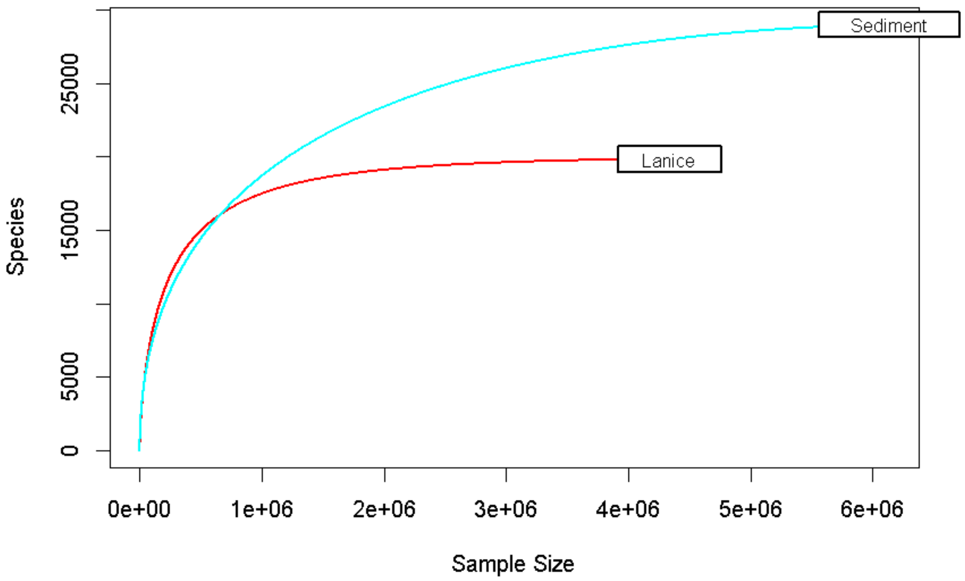

Figure 2: all *L. conchilega* samples clustered by sample type (divided by every analyzed variable). L (Lanice), M/A(may/August, Spring/ Summer), E/S (eulittoral/sublittoral), L/H(low tide/high tide), N/D (night/day). The dashed vertical lines represent the point at which 95% of the unique ASVs are found.

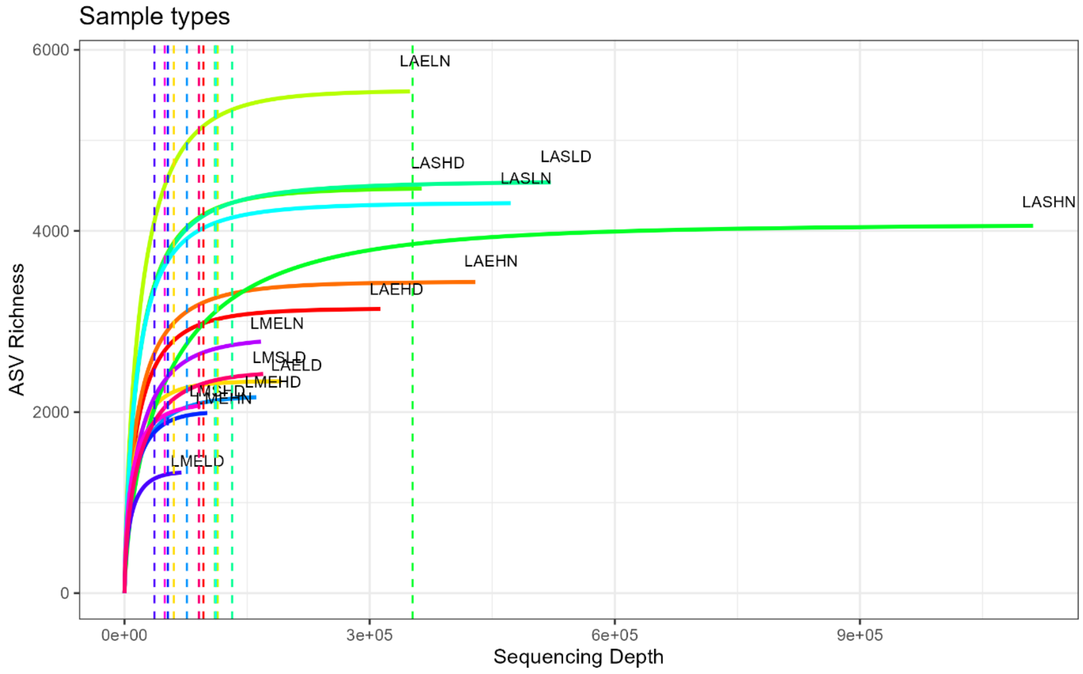

Figure 3. Zone Comparison of Alpha Diversity and Community Composition in *L. conchilega* Microbiomes. **Top, left:** Boxplots showing Simpson, Shannon, and Chao1 indices comparing *L. conchilega* microbiomes in eulittoral and sublittoral zones. The bold horizontal line indicates the median, the top and bottom of the box correspond to the 75th and 25th percentiles, and the whiskers extend to the largest and smallest values within the 1.5 interquartile range; points outside this range represent outliers. Letters (a,b) to the right of the boxes indicate statistical significance. **Top, right**: Bar plots showing the average relative abundance of every family with ≥ 10% relative abundance in at least one sample. **Bottom, left**: NMDS Ordination and Key Taya Driving Seasonal Dissimilarity by SIMPER. Top: Non-metric multidimensional scaling (NMDS) plot based on a Bray-Curtis dissimilarity matrix for *L. conchilega* samples. In dark green are the sublittoral samples; in light green are the eulittoral samples. Black arrows indicate the fitted environmental vectors of the top 10 contributing most to dissimilarity (full taxon names shown to the right). The red arrow represents the fitted vector for temperature values. **Bottom, right**: Relative abundance of the top 10 SIMPER-identified taxa contributing to dissimilarity between eulittoral and sublittoral *L. conchilega* microbiomes. P-values from permutational tests are shown on the right.

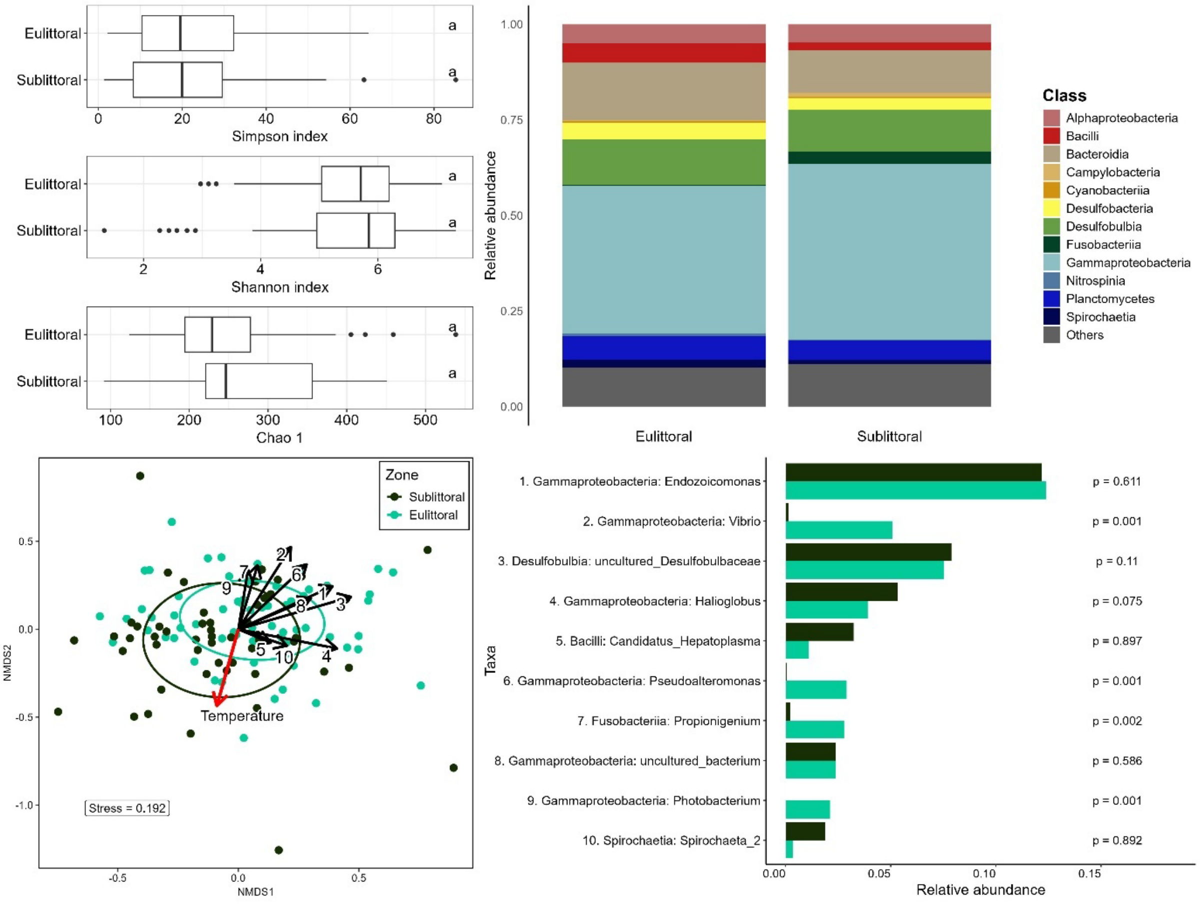

Figure 4. Tide Comparison of Alpha Diversity and Community Composition in *L. conchilega* Microbiomes. **Top, left:** Boxplots showing Simpson, Shannon, and Chao1 indices comparing *L. conchilega* microbiomes in low and high tide. The bold horizontal line indicates the median, the top and bottom of the box correspond to the 75th and 25th percentiles, and the whiskers extend to the largest and smallest values within the 1.5 interquartile range; points outside this range represent outliers. Letters (a,b) to the right of the boxes indicate statistical significance. **Top, right**: Bar plots showing the average relative abundance of every family with ≥ 10% relative abundance in at least one sample. **Bottom, left**: NMDS Ordination and Key Taya Driving Seasonal Dissimilarity by SIMPER. Top: Non-metric multidimensional scaling (NMDS) plot based on a Bray-Curtis dissimilarity matrix for *L. conchilega* samples. In orange are the low tide samples; in cyan blue are the high tide samples. Black arrows indicate the fitted environmental vectors of the top 10 contributing most to dissimilarity (full taxon names shown to the right). The red arrow represents the fitted vector for temperature values. **Bottom, right**: Relative abundance of the top 10 SIMPER-identified taxa contributing to dissimilarity between high tide and low tide *L. conchilega* microbiomes. P-values from permutational tests are shown on the right.

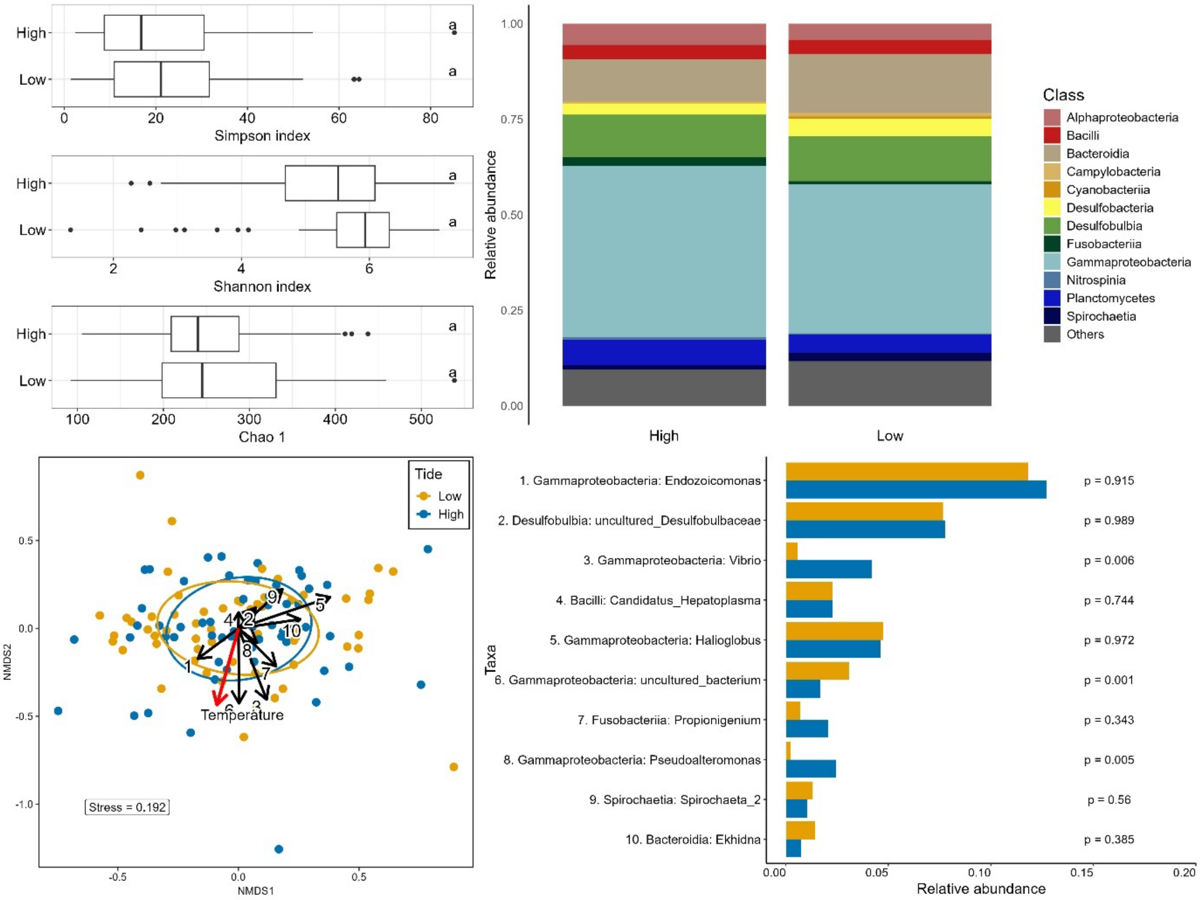

Figure 5. Time Comparison of Alpha Diversity and Community Composition in *L. conchilega* Microbiomes. **Top, left:** Boxplots showing Simpson, Shannon, and Chao1 indices comparing *L. conchilega* microbiomes in day and night. The bold horizontal line indicates the median, the top and bottom of the box correspond to the 75th and 25th percentiles, and the whiskers extend to the largest and smallest values within the 1.5 interquartile range; points outside this range represent outliers. Letters (a,b) to the right of the boxes indicate statistical significance. **Top, right**: Bar plots showing the average relative abundance of every family with ≥ 10% relative abundance in at least one sample. **Bottom, left**: NMDS Ordination and Key Taya Driving Seasonal Dissimilarity by SIMPER. Top: Non-metric multidimensional scaling (NMDS) plot based on a Bray-Curtis dissimilarity matrix for *L. conchilega* samples. In black are night samples; in light blue are day samples. Black arrows indicate the fitted environmental vectors of the top 10 contributing most to dissimilarity (full taxon names shown to the right). The red arrow represents the fitted vector for temperature values. **Bottom, right**: Relative abundance of the top 10 SIMPER-identified taxa contributing to dissimilarity between day and night *L. conchilega* microbiomes. P-values from permutational tests are shown on the right.

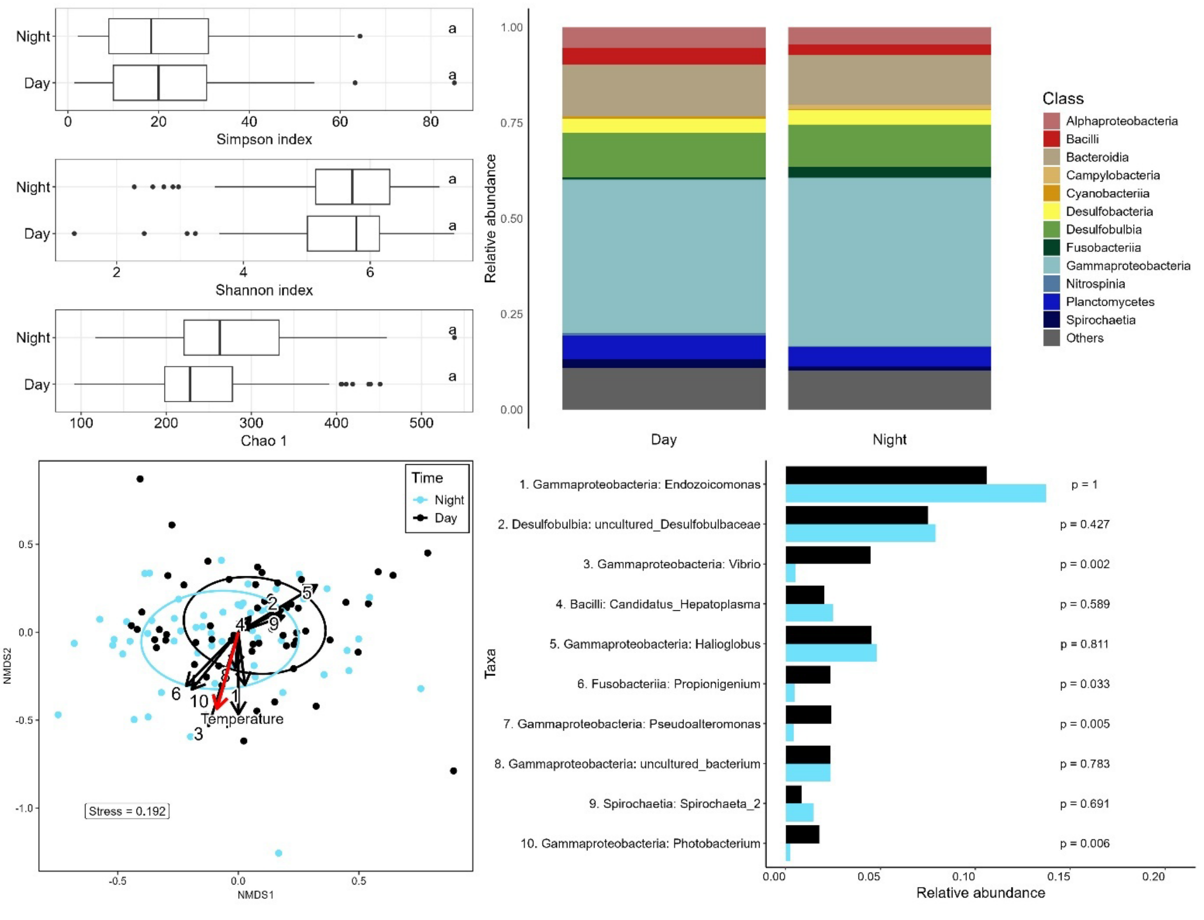

Figure 6. Heatmap showing the relative individual contribution to pathway abundance of the 10 highlighted taxa via SIMPER analysis in both spring and summer samples. On the y-axis, the predicted pathways with a Fold Change(FC) >2. On the x-axis, the top 10 SIMPER-identified taxa contributing to dissimilarity between seasonality samples. The numbers in white inside the tiles represent the relative abundance of the individual contribution of each taxon to the abundance of the highlighted predicted pathways.

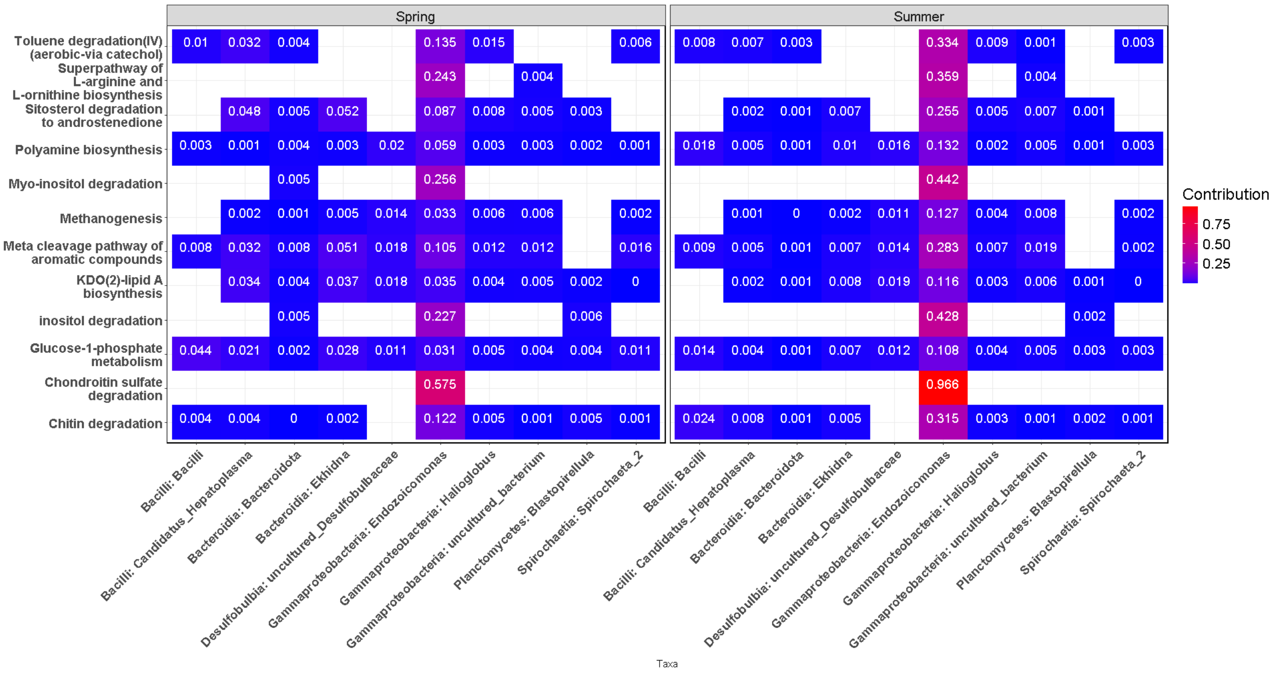

Tables

Table 1. PCR reagent table.

PCR REAGENTS	PER 25µL RXN
**5X PHUSION BUFFER**	5 µl
**10MM DNTPS**	0,5 µl
**PRIMER A**	0,75 µl each
**PHUSION DNA POLYMERASE**	0,25 µl (0.5U in 25µl = 0.02U)
**TEMPLATE DNA**	1 µl (stock: 20ng/µl)
**MOLECULAR GRADE WATER**	To 25 µl (16,25 in this case)

Table 2. PCR cycler settings

	STEP	TEMPERATURE	TIME
**1**	Initial denaturation	95°C	120s
**2**	30 cycles	95°C	45s
		50°C	45s
		68°C	90s
**3**	Final elongation	68°C	300s
**4**	Refrigeration	4°C	indefinite

Table 3. ANOVA values for all alpha diversity indices for Origin (*L. conchilega* vs Sediment)

	Inverse Simpson	Shannon	Chao1
**Df1**	1	1	1
**Df2**	239	239	239
**Sum Sq1**	1848361.723	340.0863	43537048
**Sum Sq2**	956062.4479	260.0768	40924117
**Mean Sq1**	1848361.723	340.0863	43537048
**Mean Sq2**	4000.261288	1.088187	171230.6
**F value1**	462.060248	312.5255	254.2597
**F value2**	NA	NA	NA
**Pr(>F)1**	8.97E-58	2.73E-45	1.78E-39
**Pr(>F)2**	NA	NA	NA
**THD**	1.32E-14	1.32E-14	1.32E-14

Table 4. PERMANOVA for sample Origin – *L. conchilega* vs Sediment microbiome

	Df	SumOfSqs	R2	F	Pr(>F)
**Origin**	1	8.781371	0.246448	78.16454	1.00E-04
**Residual**	239	26.85038	0.753552	NA	NA
**Total**	240	35.63175	1	NA	NA

Table 5. PERMANOVA for *L. conchilega* samples, all variables

	Df	SumOfSqs	R2	F	Pr(>F)
**Month**	1	1.602252	0.08015	10.79815	1.00E-04
**Zone**	1	0.787038	0.03937	5.304132	1.00E-04
**Tide**	1	0.534672	0.026746	3.603345	0.0003
**Time**	1	0.412798	0.02065	2.781997	0.0028
**Residual**	112	16.61879	0.831331	NA	NA
**Total**	116	19.99058	1	NA	NA

Table 6. PERMANOVA for Sediment samples, all variables

	Df	SumOfSqs	R2	F	Pr(>F)
**Month**	1	0.670993	0.098035	16.53906	1.00E-04
**Zone**	1	0.77241	0.112853	19.03886	1.00E-04
**Tide**	1	0.181199	0.026474	4.466299	0.0003
**Time**	1	0.253543	0.037044	6.249484	1.00E-04
**Residual**	119	4.827852	0.705372	NA	NA
**Total**	123	6.844409	1	NA	NA

Table 7. ANOVA values for all alpha diversity indices for Seasonality

	Inverse Simpson	Shannon	Chao1
**Df1**	1	1	1
**Df2**	115	115	115
**Sum Sq1**	9017.812481	0.063529007	655840.53
**Sum Sq2**	146841.1979	222.3298334	9792834.66
**Mean Sq1**	9017.812481	0.063529007	655840.53
**Mean Sq2**	1276.879981	1.933302899	85155.084
**F value1**	7.062380656	0.032860349	7.70171902
**F value2**	NA	NA	NA
**Pr(>F)1**	0.008991951	0.856471434	0.00644089
**Pr(>F)2**	NA	NA	NA

Table 8. ANOVA values for all alpha diversity indices for Zonation (Eulittoral vs Sublittoral)

	Inverse Simpson	Shannon	Chao1
**Df1**	1	1	1
**Df2**	115	115	115
**Sum Sq1**	12.62586	0.550535	22746.52
**Sum Sq2**	24782.67	150.1053	576620.6
**Mean Sq1**	12.62586	0.550535	22746.52
**Mean Sq2**	215.5015	1.305263	5014.092
**F value1**	0.058588	0.421781	4.536518
**F value2**	NA	NA	NA
**Pr(>F)1**	0.809172	0.517345	0.035311
**Pr(>F)2**	NA	NA	NA

Table 9. ANOVA values for all alpha diversity indices for Tide (High tide vs Low tide)

	Inverse Simpson	Shannon	Chao1
**Df1**	1	1	1
**Df2**	115	115	115
**Sum Sq1**	108.052	2.274917	1392.004
**Sum Sq2**	155751	220.1184	10447283
**Mean Sq1**	108.052	2.274917	1392.004
**Mean Sq2**	1354.356	1.914073	90845.94
**F value1**	0.079781	1.188521	0.015323
**F value2**	NA	NA	NA
**Pr(>F)1**	0.778102	0.277907	0.901702
**Pr(>F)2**	NA	NA	NA

Table 10. ANOVA values for all alpha diversity indices for Time

	Inverse Simpson	Shannon	Chao1
**Df1**	1	1	1
**Df2**	115	115	115
**Sum Sq1**	40.51951	0.011027	12304.82
**Sum Sq2**	24754.78	150.6448	587062.3
**Mean Sq1**	40.51951	0.011027	12304.82
**Mean Sq2**	215.2589	1.309955	5104.89
**F value1**	0.188236	0.008418	2.410398
**F value2**	NA	NA	NA
**Pr(>F)1**	0.665202	0.927057	0.123279
**Pr(>F)2**	NA	NA	NA
